# CYP26A1 Is a Novel Biomarker for Betel Quid-Related Oral and Pharyngeal Cancers

**DOI:** 10.3390/diagnostics10110982

**Published:** 2020-11-21

**Authors:** Ping-Ho Chen, Chia-Min Chung, Yen-Yun Wang, Hurng-Wern Huang, Bin Huang, Ka-Wo Lee, Shyng-Shiou Yuan, Che-Wei Wu, Lee-Shuan Lin, Leong-Perng Chan

**Affiliations:** 1 School of Dentistry, College of Dental Medicine, Kaohsiung Medical University, Kaohsiung 80708, Taiwan; phchen@kmu.edu.tw (P.-H.C.); wyy@kmu.edu.tw (Y.-Y.W.); 2 Institute of Biomedical Sciences, National Sun Yat-sen University, No. 70 Lienhai Road, Kaohsiung 80424, Taiwan; hwinlife@gmail.com; 3 Center for Cancer Research, Kaohsiung Medical University, Kaohsiung 80708, Taiwan; yuanssf@kmu.edu.tw; 4 Cancer Center, Kaohsiung Medical University Hospital, Kaohsiung Medical University, Kaohsiung 80708, Taiwan; 5 Cohort Research Center, Kaohsiung Medical University, Kaohsiung 80708, Taiwan; cwwu@kmu.edu.tw; 6 Center for Drug Abuse and Addiction, China Medical University Hospital, China Medical University, Taichung 406040, Taiwan; ibmsn141@gmail.com; 7 Graduate Institute of Biomedical Sciences, China Medical University, Taichung 406040, Taiwan; 8 Department of Biomedical Science and Environmental Biology, College of Life Science, Kaohsiung Medical University, Kaohsiung 80708, Taiwan; huangpin2@kmu.edu.tw; 9 Regenerative Medicine and Cell Therapy Research Center, Kaohsiung Medical University, Kaohsiung 80708, Taiwan; 10 Department of Otorhinolaryngology-Head and Neck Surgery, Kaohsiung Municipal Ta-Tung Hospital and Kaohsiung Medical University Hospital, Kaohsiung 80708, Taiwan; kawolee@kmu.edu.tw; 11 Department of Medical Research, Kaohsiung Medical University Hospital, Kaohsiung 80708, Taiwan; 12 Graduate Institute of Medicine, College of Medicine, Kaohsiung Medical University, Kaohsiung 80708, Taiwan; 13 Translational Research Center, Kaohsiung Medical University Hospital, Kaohsiung 80708, Taiwan; 14 Department of Obstetrics and Gynecology, Kaohsiung Medical University Hospital, Kaohsiung 80708, Taiwan; 15 Department of Otorhinolaryngology-Head and Neck Surgery, Kaohsiung Municipal Siaogang Hospital, Kaohsiung Medical University, Kaohsiung 80708, Taiwan; 16 Faculty of Medicine, College of Medicine, Kaohsiung Medical University, Kaohsiung 80708, Taiwan; 17 Laboratory of Veterinary Diagnostic Imaging, Department of Veterinary Medicine, College of Veterinary Medicine, National Pingtung University of Science and Technology, Pingtung 912301, Taiwan; linleeshuan@gmail.com

**Keywords:** betel quid, oral and pharyngeal cancers, CYP26A1, arecoline

## Abstract

Betel quid (BQ) has been classified as a Group I human carcinogen in light of evidence demonstrating an association with an elevated risk of oral and pharyngeal cancers. To date, the incidence rate of oral and pharynx cancers among Taiwanese men ranks the highest worldwide. However, no study has yet confirmed variants of CYP26A1 was associated with the risks of oral and pharyngeal cancers. A case-control study was conducted (n = 339). CYP26A1 polymorphism was performed using SNP assay. Real-time qRT-PCR and Western blotting were used to determine the levels of CYP26A1 expression. The cancer cell model involved treatment with arecoline. Our findings showed that the downregulation of CYP26A1 mRNA and protein expression are more frequently observed in cancerous tissues than adjacent normal tissues in patients with oral and pharynx cancers (*p* < 0.01). We found that CYP26A1 was downregulated as the arecoline dose increased. We hypothesized that lower levels of CYP26A1 mRNA expression can be utilized a clinically biomarker causes oral and pharynx cancers. Arecoline appears to modulate CYP26A1 expression through specific pathways. Carriers of CYP26A1 SNP, rs2068888 (G/G)/rs4418728 (G/G) and who have lower levels of CYP26A1 expression are associated with an increased risk of oral and pharyngeal cancers.

## 1. Introduction

Approximately 600 million people worldwide chew betel quid (BQ), a habit that is strongly associated with oral and pharyngeal cancers [1,2]. A positive correlation has been demonstrated between BQ chewing rates and the incidence oral and pharyngeal cancers in different countries [3]. In Taiwan, it is estimated that about over two million people chew BQ, composed mainly of men (15.6% prevalence rate for men compared to 3.0% rate for women) [4,5]. In 2017, the cancer registry annual report indicated that oral cavity and pharynx cancers had the fourth highest incidence of cancers among Taiwan males, as well as ranking as the fourth leading cause of death due to cancer. The age standardized incidence rate (adjusting by the 2000 world standard population) was 41.15 people, and the mortality rate was 15.04 per 100,000 persons, respectively [6].

Previous studies have suggested that increased ingestion of retinoic acid (RA) reduces the carcinogenesis at different sites (such as oral, skin, breast, lung, laryngeal, cervical, bladder, prostate, and ovarian cancers) [7,8]. A critical review indicated that low levels of RA in human serum may increase the risk of developing certain types of cancer [9,10]. Moreover, an enhanced risk of cancer was found in vitamin A deficient animals [8,10,11]. RA is also known as all-*trans*-retinoic acid (ATRA), tretinoin, and vitamin A [11]. In animal models, vitamin A deficiency increases susceptibility to chemical carcinogens and is associated with a higher risk of cancer incidence [10]. RA is a biologically active derivative of vitamin A and participates in different cell processes, including growth, differentiation, and apoptosis [12]. In particular, RA plays a crucial role in the growth regulation and differentiation of normal, premalignant, and malignant cell types, especially epithelial cells, and this role is primarily mediated via its interaction with two types of nuclear RA receptors: retinoic acid receptors (RARs) and retinoic acid X receptors (RXRs) [10].

The effects of cellular RA metabolism, through the regulation of RA-targeted genes (CYP26 family) that are mediated by specific RA receptors, are mainly due to the mediation of RA receptors (RARs and RXRs) regulation. Furthermore, altered RA receptor expression may be related to the malignant transformation of cultured human cells or animal tissue [10]. The CYP26 family is composed of three isoforms, namely, CYP26A1, CYP26B1, and CYP26C1; both CYP26A1 and CYP26B1 play major roles in the regulation of cellular RA metabolism [13]. Previous studies have suggested that the occurrence of cancer in humans may be closely related to aberrations in the balance signal for RA, primarily via the dysregulation of RARs and RXRs [10,14].

The imbalance in RA signaling may be associated with occurrence of oral and pharyngeal cancer, and the low expression of RARs, lower levels of cellular RA, reduced transcription of RA-metabolizing enzyme CYP26A1, and changes in RA metabolism may be involved in the carcinogenic pathway [15]. The carcinogenesis of oral and pharyngeal cancers may be due to oral mucosal cell hyperplasia and hyperkeratosis induced by RA deficiency [7]. Therefore, we hypothesize that the CYP26A1 expression may be associated with RA metabolism via RA receptors, and that lower RA levels may increase the risk of oral and pharyngeal cancers.

Our previous studies reported that CYP26B1 variants are associated with oral and pharynx cancers and that the expression of the CYP26B1 spliced variant may be related to oral and pharynx cancers [2,16]. However, to the best of our knowledge, the expression of CYP26A1 in oral and pharyngeal tumor tissues compared to their adjacent normal tissues in humans has not yet been reported in oral and pharynx cancer studies. Therefore, this study was designed to determine whether CYP26A1 variants play an important role in determining the risk of oral and pharyngeal cancer occurrence.

## 2. Materials and Methods

### 2.1. Study Subjects and Specimens

A hospital-based case control study was designed and approved by the Research and Ethical Review Committees at the Kaohsiung Medical University (KMU) Hospital (Institutional Review Board (IRB) no. KMUH-IRB-20110031, KMUHIRB-20130076, KMUHIRB-20140033, and KMUHIRB-20140142). Patients were collected from the Department of Otolaryngology and Division of Oral and Maxillofacial Surgery, Department of Dentistry in KMU Hospital.

The aims and procedures of the study were explained to all the volunteer participants. Written informed consent was obtained from all participants prior to data collection, including agreement to interviews by trained interviewers and the provision of blood specimens and resected tissues of cancerous and adjacent non-cancerous (safety margin) oral and pharyngeal tissues during surgery resection for experimental analysis. Tissue samples were collected during the necessary surgery procedures, for use in CYP26A1 gene expression analysis.

Out of a total of 339 subjects, 83 patients (81 males) were diagnosed with oral and pharyngeal cancers, and the control group consisted of 256 unrelated healthy controls (245 males). Among the 83 patients, we identified only 37 patients (35 males) with paired oral and pharyngeal tissues. Most of our patients were males due to BQ chewing habits and because most oral and pharyngeal patients in Taiwan are men. This observation is line with other reports [5,6].

All cases were histologically confirmed to have oral and pharyngeal cancers by surgeons and pathologists. All of the subjects were interviewed by trained interviewers using a standardized questionnaire, which included question on personal demographic data, clinical characteristics, and previous history of substance use (in particular for BQ). Lifetime BQ chewers were denoted as any individual who chewed BQ at least 1 quid of any type of BQ per day for a minimum of six months. According to a previous study [17], any individual who chewed at least 1 quid of any type of BQ per day for a minimum of six months was defined as a lifetime BQ chewer [17].

### 2.2. CYP26A1 Genotyping

Genomic DNA was extracted from peripheral blood lymphocytes. The CYP26A1 genotyping was performed using the TaqMan Single Nucleotide Polymorphism (SNP) assay. The reaction plates were then read using the ABI Prism 7900HT Sequence Detection System. The fluorescence results were analyzed and auto called into genotypes using the built-in software of the system. In the present study, we chose two previously reported single nucleotide polymorphisms (SNPs) (*CYP26A1* rs2068888 [18] and rs4418728 polymorphism [19]). Moreover, these two SNPs of the downstream of CYP26A1 were selected because they have minor allele frequencies (≥5%) in healthy controls from the public database: Chinese HapMap-CHB.

### 2.3. Cell Viability Assay

Human Ca9-22, SAS, HSC-3, and CAL 27 oral cancer cell lines were purchased from American Type Culture Collection (ATCC) (Manassas, VA, USA) and Health Science Research Resources Bank (HSRRB) (Osaka, Japan) [20].

The cell culture method and MTT assay were used to estimate the cell viability (%) after exposure to oral cancer cells at different concentrations of arecoline for 24 h, as described previously [21]. Briefly, the medium supplemented with arecoline (0, 100, 200, 400, and 800 μM) was freshly prepared by adding arecoline hydrobromide (Sigma) to a growth medium (DMEM/F12). The cytotoxicity was expressed as the concentration of arecoline inhibiting cell growth by 50% (IC_50_). The Ca9-22 cells were treated with various concentrations of arecoline (0, 100, 200, 400, and 800 μM) for 24 h, and SAS cells were treated with various concentrations of arecoline (0, 100, 200, 400, 600, and 800 μM). Additionally, HSC-3 cells were treated with an IC_50_ values of 400 μM arecoline, while CAL 27 cells were treated with IC_50_ values of 100 μM arecoline.

The MTT assay was used to evaluate cell proliferation for 2 h in a CO_2_ incubator at 37 °C. Using an ELISA reader (Bio Tek el800), the cells were treated with DMSO, followed by the measurement of the absorbance (570 nm) using 630 nm as a reference after the removal of the culture medium. The percentage of viable cells was determined in comparison with the vehicle controls.

### 2.4. Reverse Transcription

To prepare a cDNA pool from each RNA sample, 5 µg of total RNA were reverse transcribed using MMLV reverse transcriptase (Promega). The resulting samples were diluted 40-fold with DNase-free water. Each cDNA pool was stored at −20°C until further analysis by real-time PCR.

### 2.5. PCR Primers

Specific oligonucleotide primer pairs were selected from Roche Universal ProbeLibrary for the real-time PCR assays. The specificity of each primer pair was validated by RT-PCR using a common reference RNA (Stratagene, La Jolla, California, USA) as the DNA template. The size of the PCR product was checked by a DNA 1000 Chip (Agilent Technologies, Santa Clara, California, USA) run on a Bioanalyzer 2100 (Agilent Technologies, USA). The primer pairs that generated the predicted product size without any other side products were selected for subsequent real-time RT-PCR reactions.

### 2.6. Real-Time PCR

Real-time PCR was performed using a LightCycler Instrument 1.5 (Roche) and LightCycler^®^ FastStart DNA Master^PLUS^ SYBR Green I kit (cat. no. 03 515 885 001; Roche, Castle Hill, Australia). Briefly, 10 µL of sample, comprised of 2 µL of Master Mix, 2 µL of 0.75 µM forward and reverse primers, and 6 µl of cDNA sample, were run. Each sample was run in triplicate using the following program: 95 °C for 10 min, 40 cycles of 95 °C for 10 s, 60 °C for 15 s, and 72 °C for 10 s. At the end of the program, melting curve analysis was performed. At the end of each RT-PCR run, the data were automatically analyzed by the system, and an amplification plot was generated for each cDNA sample. From each of these plots, the LightCycler3 Data analysis software automatically calculated the Ct (crossing point) value, which denotes the turning point corresponds to the first maximum of the second derivative curve, used to indicate the beginning of exponential amplification. The mRNA fold expression or repression of the target gene relative to the internal control gene GAPDH in each sample was then calculated using the 2^−△△Ct^ method, based on the following formula:

△Ct = C_t target gene_ − C_t internal control_

△△C_t_ = △C_t test sample_ − △C_t control sample_

This method was used to analyze the relative gene mRNA expression through real-time quantitative PCR.

### 2.7. Protein Extraction and Western Blotting

The samples were rinsed with wash buffer before sonicating with lysis buffer to obtain the cell lysate samples. The protein concentration was assayed using the BCA protein assay reagent (Thermo Fisher Scientific Inc., Rockford, IL, USA). The cell lysate was mixed with sodium dodecyl sulfate polyacrylamide gel electrophoresis (SDS-PAGE) sample buffer, and then separated by SDS-PAGE. The blotted membranes were hybridized using rabbit monoclonal for CYP26A1 (ab172474; Abcam) and anti-β-actin antibody. The membranes were then incubated with SuperSignal West Femto reagent (Thermo Fisher Scientific Inc.) and exposed to X-ray films for the indicated time period. Lastly, a digital scanner (Microtek International Inc., Hsinchu, Taiwan) was used to obtain images of the samples on X-ray film. Three replicates were scanned and analyzed for each sample. Progenesis Samespots v2.0 software (NonLinear Dynamics) was used to estimate the levels of CYP26A1 protein expression. β-actin was used to normalize the protein levels.

### 2.8. Statistical Analysis

The mean, standard deviation (SD), and percentage (%) were used to describe the distribution of the demographic factors of the participants, including the age at first use and the amount and duration of substance use (alcohol, betel quid, and cigarette). We used chi-square test or Fisher’s Exact test (when expected values were less than 5 or above expected values of 20% cell were less than 5) to compare the distribution of the category data. The mRNA and protein expression variables were presented as medians and inter quartile range (IQR) of data that were not normally distributed. Non-parametric Wilcoxon signed-rank test was used to analyze the differences in CYP26A1 expression. The application performance of the distinguishability of CYP26A1mRNA expression between oral and pharynx sites was determined by the receiver operating characteristic (ROC) curve. Maximized Youden’s index (sensitivity + specificity − 1) was used to determine the optimal cut-off points of mRNA scores for oral sites and pharynx sites between low and high mRNA levels [22]. All statistical analyses were carried out using SPSS version 20 (SPSS Institute Inc., Chicago, Delaware, USA) and the SAS Statistical Package (Version 9.4, SAS Institute Inc., Cary, North Carolina, USA). Results denoted by asterisks were considered to be statistically significant (two-sided *p*-values <0.05, <0.01 or <0.001).

### 2.9. Sample Size Calculation and Power of Study

G∗Power (version 3.1.9.4) (program written, conceptualized, and designed by Franz, Universität Kiel, Germany) is a freely available Windows application software and was used for power estimation. For gene expression analysis, pairs of cancer tissue and its adjacent non-cancerous tissue were obtained from oral and pharyngeal cancer patients. Based on the non-parametric Wilcoxon signed-rank test, with an estimated “medium” effect size of 0.50, and a type I (α) error of 0.05, a total of 37 participants were recruited for this study. Post hoc power analyses indicated that the post hoc power was at least 82%, and a type II error (β) of 0.18 was present.

Our study design was a case-control study. In general, a large sample size can increase statistical power and significance of a study. A case-control study may add the sample case and control sizes to obtain sufficient statistical power. Nevertheless, due to various reasons, increasing the cases sample size is not always feasible. Particularly, under some circumstances, the sample size of cases cannot be increased favorably. For instance, in case of rare diseases, the efforts required to enroll controls is lower than the efforts required to enroll cases than in other diseases. Epidemiologic textbooks recommend that researchers plan a case-control study to collect no more than four controls per case (controls: cases ≤ 4:1) because increasing this ratio rarely increases the statistical power of the study [23,24]. During the subject enrollment process, cost and feasibility become important considerations. There are settings in which a higher control-to-case ratio may be desirable. Based on this rationale, we used a higher ratio of controls-to-cases (3:1) study design to increase statistical power in this study [25].

In addition, based on the Chi-square test, some differences are clearly visible, mainly concerning cigarette smoking, alcohol consumption, and genetic factors. In this study, we recruited 339 patients to reach a statistical power of at least 95%, with an estimated effect size of 0.36, and an α of 0.05. Additionally, logistic regression analysis was performed to examine the association between risk of oral and pharyngeal cancers, and environmental factors and genetic SNP in a multivariate model. Therefore, a multivariate logistic model can adjust the effect of confounding factors (such as BQ chewing, cigarette smoking, and alcohol drinking) to assess these factors and compare their effects to elucidate whether genetic background predisposes people toward cancer risk.

## 3. Results

A total of 339 study participants were included, of whom 97.6% were male; the proportion of gender was not significant different in oral and pharyngeal cancers (97.6%) and controls groups (95.7%) (*p* = 0.437). The mean age of the oral cancer cases and control were 58.9 ± 9.3 years and 44.7 ± 12.8 years, respectively (*p* < 0.001). Significant differences were noted in the marriage status, age group (less than 50 or large than 50) the habit of drinking alcohol, BQ chewing and cigarette smoked, but no significant differences in education level were observed between cases and controls (Table 1). Genotyping analysis results are presented in Table 1. Two SNPs, rs2068888 and rs4418728, in the downstream of CYP26A1 were genotyped to examine association with the occurrence of oral and pharyngeal cancers. For all 2 SNPs, there was no deviation from a Hardy–Weinberg equilibrium in either the cases or the controls. These two SNPs were significantly associated with risk of oral and pharyngeal cancers (*p* = 0.009) and complete linkage disequilibrium (Table 1).

We selected rs2068888 as tag SNP to examine associations with the risk of oral and pharyngeal cancers. The univariate associations of environmental factors and genetic SNP with oral and pharyngeal cancer risk are presented in Table 2. We found that marriage status, age group (less than 50 or large than 50) the habit of drinking alcohol, BQ Chewing and cigarette smoked and genetic SNP were associated with oral and pharyngeal cancer risk. Subjects carrying variant G/G genotype of rs2068888, had an increased risk of oral and pharyngeal cancer risk (OR = 5.8, 95% CI = 1.7–20.3); Using logistic multivariable model, the habit of drinking alcohol and cigarette smoked were not significantly associated with the risks of oral and pharyngeal cancer (Table 2).

In order to evaluate the interaction between genetic SNP and the use of BQ, we categorized genetic SNP and the use of BQ chewing in either of two categories (risk allele: yes/ no; use: yes/ no). In terms of their causal effects, two risk factors may act independently or may interact with each other, thereby augmenting (in case of synergism) or reducing (in case of antagonism) the effect of each another. Table 3 shows the joint effects of genetic SNPs and BQ use in oral and pharyngeal cancer patients. We found that OR of genetic SNP increased with the habit of BQ Chewing compared to controls without BQ chewing. Odds ratio increased from 5.4 to 16.2. The synergy index was 0.98 (95% CI: 0.60–1.10). The synergy index was close to 1. It means that genetic SNP and BQ chewing had a joint effect on the risk of oral and pharyngeal cancers in an additive manner.

However, the relatively low frequency in the population of the G/G genotypes (0.8% among controls) and of the joint exposure (4.8%) translates into considerable population attributable fractions for oral and pharyngeal cancers (3.3% and 4.5%, respectively; Table 3). The demographic characteristics, substance use habits, and clinical factors (such as cancer site, TNM stage, morphologic type, and treatment modality) of patients with oral and pharyngeal cancers were shown in supplementary table (Table S1).

We obtained paired oral and pharyngeal tissues, as well as adjacent normal tissues (Table S1) from a total of 37 patients. The data in Table S1 shows the sociodemographic characteristics and alcohol, BQ consumption, and smoking habits of the enrolled patients. A number of patients did not complete the questionnaire and are presented as missing data in the table. No significant differences (*p* > 0.05) were observed between the oral tumor site patients and pharyngeal tumor site patients in terms of distribution. A total of 80.6% of the patients were Hokkien in ethnicity. Their average age was 53.5 ± 8.8 years, where 71.0% of patients had a low education level (≤9 years). Among these patients, 80.6% drank alcohol regularly, 84.8% chewed BQ, and 92.6% smoked. The average age for starting to chew BQ was 22.6 ± 6.2 years old, with an average time period of BQ consumption of 31.9 ± 10.0 years. The daily average consumption of BQ was 26.0 ± 13.6 quids, with a cumulative BQ exposure of 81.0 ± 44.1 (pack × year) for males with oral and pharyngeal cancers. The cumulative BQ exposure (pack ‒ years) was calculated as the number of packs (number of betel quid/10) consumed multiplied by the years of BQ consumption. One pack was defined as 10 quids.

**Figure 1:**
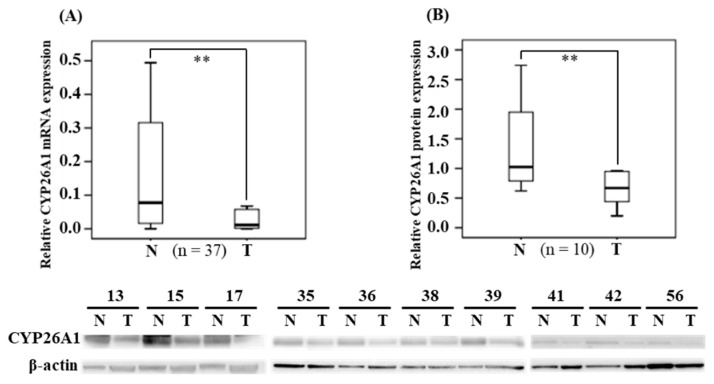
Expression of CYP26A1 in human oral and pharyngeal cancer tissues. (**A**). The relative mRNA (2^−ΔΔCt^) expression of CYP26A1 (n = 34) in human oral and pharyngeal tumor (T) tissues compared with their adjacent normal (N) tissues. (**B**). In human oral and pharynx tumor (T) tissues compared with their adjacent normal (N) tissues (representative cases are shown with *β-actin* as the protein loading control), the protein levels were examined by Western blotting and quantitated using Image J (n =10). The results are represented as the median (IQR). The boxes denote the interquartile ranges, while the bars represent the highest and lowest values, excluding outliers and extreme outliers. ** *p* < 0.01 compared to the normal group.

To exclude the individual differences in CYP26A1 expression, we collected oral and pharynx cancerous tissues and their paired tissues (adjacent non-cancerous tissues), which were used as the controls, from 37 patients. The median (IQR) (median = 0.011; IQR = 0.002–0.058) expression of CYP26A1 mRNA in cancerous tissues was significantly lower than in the adjacent non-cancerous normal tissues (median = 0.077; IQR = 0.014–0.316) (*p* = 0.002 < 0.01), according to non-parametric Wilcoxon signed-rank testing (Figure 1A). Subsequently, in BQ chewers (n = 28), we compared the expression of CYP26A1 mRNA in human oral sites and pharynx tumor tissues, respectively (Figure S1). We found that the median (IQR) (median = 0.016; IQR = 0.003–0.105) expression of CYP26A1 mRNA in pharynx tumor tissues was found significantly lower than at oral tumor sites (median = 0.067; IQR = 0.019–0.254) (*p* = 0.036 < 0.05). However, in adjacent normal tissues, there were no statistical significant differences between human oral and pharyngeal tumor sites. Western blotting analysis confirmed the levels of CYP26A1 protein expression and indicated that the protein levels in cancerous tissues were significant lower than in the adjacent non-cancerous tissue (*p* = 0.007 < 0.05) (Figure 1B). The results for the CYP26A1 protein levels are consistent with the levels of CYP26A1 mRNA expression.

**Figure 2:**
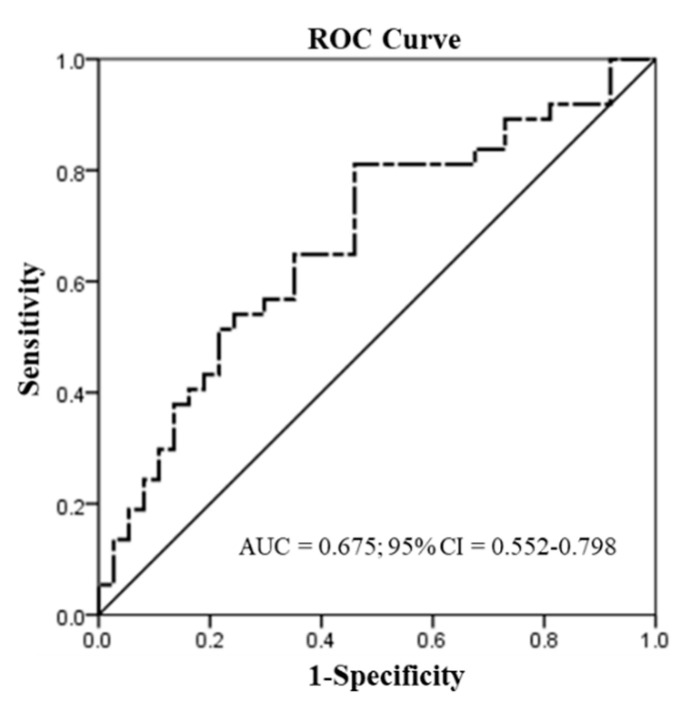
Receiver operating characteristic (ROC) curve analysis of the 2^−ΔΔCt^ scores of mRNA in human oral and pharyngeal tumor (T) tissues (n = 37). The area under the curve (AUC) = 0.675 (95% confidence interval (CI) = 0.552–0.798; *p* = 0.01 < 0.05) was used to differentiate the 2^−ΔΔCt^ scores <0.069 for tumor tissues from the 2^−ΔΔCt^ scores ≥0.069 for adjacent normal tissues.

We used the ROC curve to predict the discriminatory power for patients with oral or pharyngeal site tumors (n = 37). In the assessment of CYP26A1 mRNA, the mRNA levels were used to distinguish the tumor and adjacent normal tissues (Figure 2). A significant application performance (area under the curve (AUC) = 0.675; 95% confidence interval (CI) = 0.552–0.798; *p* = 0.01 < 0.05) was demonstrated by the ROC curve for the mRNA levels of CYP26A1 between tumor and adjacent normal tissues. Maximized Youden’s index was used to determine the optimal cut-off points of mRNA scores for the prediction of tumor and adjacent normal tissues. Indeed, we found the best discrimination of mRNA levels < 0.069 (tumor) and mRNA levels ≥ 0.069 (normal) between tumor and adjacent normal tissues.

**Figure 3:**
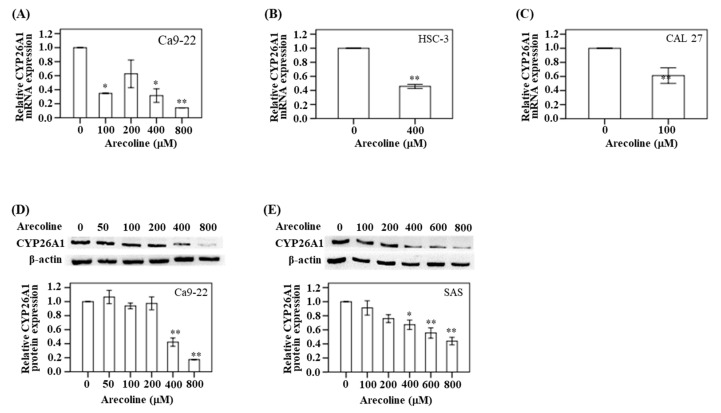
CYP26A1 expression in oral cancer cells treated with different concentrations of arecoline. (**A**) The relative mRNA expressions were detected in Ca9-22 treated with arecoline (0, 100, 200, 400, and 800 μM). (**B**) HSC-3 treated with arecoline (0 and 400 μM). (**C**) CAL 27 treated with arecoline (0 and 100 μM). (**D**) The relative protein expressions were detected in Ca9-22 treated with arecoline (0, 50, 100, 200, 400, and 800 μM). (**E**) The relative protein expressions were detected in SAS treated with arecoline (0, 100, 200, 400, 600, and 800 μM). The average fold change (mean ± SD) of the CYP26A1 gene was measured in triplicate. * *p* < 0.05 and ** *p* < 0.01 compared with untreated control.

As mentioned above, we observed lower levels of CYP26A1 mRNA expression in the oral and pharynx tumor tissues. We used a cell model to confirm this finding. In Ca9-22 cells, the expression of CYP26A1 mRNA was assayed after treatment with five different concentrations of arecoline (0, 100, 200, 400, and 800 μM) for 24 h. Compared with the untreated control group, the CYP26A1 mRNA expression was found to decrease in the Ca9-22 cells as the arecoline concentration increased. This was particularly significant at 800 μM arecoline (*p* < 0.01) (Figure 3A). In addition, Western blotting analysis demonstrated a downregulation of protein expression at higher arecoline doses (400 and 800 μM) (Figure 3D). Similarly, the change in downregulation of CYP26A1 protein was particularly significant (*p* < 0.05) at 400, 600, and 800 μM arecoline treatment in SAS cell (Figure 3E). In HSC-3 cells, a significant downregulation in CYP26A1 mRNA expression was observed after treatment with 400 μM arecoline (*p* < 0.05) compared with the untreated control group (Figure 3B). Treatment with 100 μM arecoline resulted in a decreased expression of CYP26A1 mRNA in CAL 27 cancer cells (*p* < 0.05) compared with the untreated control group (Figure 3C).

## 4. Discussion

In this study, we found that the downregulation of CYP26A1 expression may play an important role in the occurrence of oral and pharyngeal cancers. Our results have revealed an important insight into the impact of BQ chewing on the two single nucleotide polymorphisms (SNPs), rs2068888 or rs4418728, of CYP26A1, and oral and pharyngeal cancer risk (rs2068888, odds ratio (OR) = 5.0; 95% CI = 1.1–22.1). Our findings were consistent with those of previous studies with regards to the significant downregulation of CYP26A1 in hepatocellular carcinoma tissue compared to paired-matched non-tumor tissue, using quantitative mRNA expression analysis [26]. We hypothesized that the lower levels of CYP26A1 expression in oral and pharyngeal cancer tissue may be due to lower RA levels, reduced retinoid signaling due to RXR phosphorylation, or an unidentified factor that may be specifically expressed in tumor tissues. A previous study found that the levels of CYP26A1 mRNA expression in human epidermal keratinocytes were low [27]. Similarly, in a mouse model (retinol dehydrogenase 1 (rdh1)-null mouse), the low expression of CYP26A1 mRNA was found to compensate for damaged rdh1 [28]. In the case of RA deficiency, the long-term downregulation of CYP26A1 may be critical for the conservation of RA, while an acute upregulation of CYP26A1 may be important for preventing excessive levels of RA, derived from either exogenous sources or dietary food [29]. In contrast, a previous study have found that CYP26A1 expression is upregulated in colorectal cancer [30].

CYP26A1 is known to participate in the regulation of the cellular retinoic acid (RA) metabolism. RA is a biologically active derivative of vitamin A and is regulated by different cell processes, including growth, differentiation, and apoptosis. It is known to play an important role in visual physiological function, embryonic development patterns, and adult physiological mechanisms [10,13]. RA requires an appropriate balance mechanism to control its concentration [10,13]. The CYP26 family is partially responsible for the regulation of intracellular retinoid compounds (such as all-trans-retinoic acid (at-RA)) via metabolic oxidation process, indicating that it may influence the messaging and homeostasis mechanism of RA [13]. In an earlier study, high levels of CYP26A1 mRNA expression were found to be induced by RA in certain human tumor cell lines, further indicating that the RA-inducible RA metabolism may be associated with CYP26A1 expression [31]. A previous study found that the expression of CYP26A1 may be induced by the RA receptors in breast and colon cancer cells [32]. Moreover, high levels of CYP26A1 expression and RA catabolic activity have been detected in breast epithelial adenocarcinoma tissue culture, head and neck cancer squamous cells, and acute promyelocytic leukemia cells [33,34].

Previous studies have shown that the CYP26 families (such as CYP26A1) and its related RA receptors (RARs) and retinoid X receptors (RXRs) regulate the metabolic mechanism of RA. This has an effect on the retinoic acid balance signal and may thus be associated with the development of cancer [35]. A previous study reported that the miR-34a-dependent downregulation of RXRα decreased the induction of downstream CYP26 genes of RXRα [36]. Another suggested that the occurrence of human cancers may be closely related to a lack of retinoic acid balance as a result of interactions with two types of nuclear receptors: retinoic acid receptors (RARs) and retinoid X receptors (RXRs) [10]. The lack of a normal RA signaling may result from a low expression of RARs, which reduces RA transcription while increasing the RA metabolism [15].

In clinical tissues, we found a significant downregulation of CYP26A1 in oral and pharyngeal cancer tissues compared with their adjacent tissues. Since 89.3% of the enrolled patients actively chewed BQ, we hypothesized that chewing BQ may result in an alteration to the RA metabolism through the induction of CYP26A1 expression, in the oral mucosa. The hypermethylation of RAR-β, resulting in the loss of expression, may be implicated in arecoline-related oral carcinogenesis [37]. A previous study indicated that RAR-β expression was found in 100% of normal oral tissues, but only 40% (21/52) of RAR-β expression in oral potential malignant disorder tissues, with statistically significant differences (*p* = 0.003) [38]. Other studies have also pointed out that the loss of RAR-β expression and the excessive expression of RAR-α are significantly associated with the development of oral cancers [39,40]. In contrast, one study pointed out that the expression of RARs and RXRs in oral cancer tissues was higher than that in dysplastic oral mucosa and normal tissues [41]. As such, the association between CYP26A1, RARs, RXRs, and RA levels will need to be elucidated in future studies.

In Figure S2, the expression levels for G/G genotypes (n = 1) of rs2068888 in the CYP26A1 were lower compared to A/A (n = 12) and G/A genotypes (n = 5) of rs2068888. The statistic was not significant, due to only one sample in the G/G genotypes group. This study is the first to demonstrate that lower levels of CYP26A1 expression are significantly associated with the risk of oral and pharyngeal cancers, showing a relationship between the levels of CYP26A1 and SNP polymorphism. In order to exclude the differences in CYP26A1 expression between individuals, we used the same patients with paired tissue to confirm the association between CYP26A1 expression and the occurrence risk of oral and pharyngeal cancers.

### Study Limitation

Our study design was a case-control study. All patients with oral and pharyngeal cancer were diagnosed by clinicians and pathologists. The patients were enrolled by purposive sampling and identified by clinicians at the Department of Otolaryngology and Division of Oral and Maxillofacial Surgery, Department of Dentistry, KMU Hospital. However, the study has as a limitation that it is prone to certain biases, especially selection bias. If cases and controls are not chosen similarly from the base of the study, it leads to selection. Further, selection bias can occur during the identification of case patients and controls if their inclusion relies on their interest exposure. In addition, our study has a small tissue sample size. Although our sample size of cancer tissues was only 37, the power estimation of this study was 82% (>optimal values of 80%). As such, our results have a 82% valid probability of being significant. This high statistical power indicates that our test results are most likely valid.

## 5. Conclusions

In conclusion, we demonstrated that carriers of downregulated CYP26A1 expression are susceptible to an increased risk of oral and pharyngeal cancers and the joint effects of genetic SNPs (rs2068888 (G/G) or rs4418728 (G/G)) and BQ use in oral and pharyngeal cancer patients. We also found that arecoline can modulate the expression of CYP26A1. We suggest that CYP26A1 plays a novel biomarker in modulating the BQ dependent pathogenic effect on the risk of oral and pharyngeal cancers (Figure S3), particularly individuals with at-risk genotype and lower expression (mRNA levels < 0.069) of CYP26A1.

## Figures and Tables

**Table 1:** Characteristics of the oral and pharyngeal cancers and controls (n = 339).

Characteristics	Cases		Controls		
	n = 83		n = 256		*p* Value
Male, N (%)	81	(97.6) ^b^	245	(95.7)	0.437 ^a^
Diagnostic age (yrs), mean ± SD	58.9 ± 9.3		44.7 ± 12.8		<0.001 ^c,^*
Age group, N (%)					
Age ≤ 50	15	(18.1)	178	(69.5)	<0.001 *
Age > 50	68	(81.9)	78	(30.5)	
Marriage, N (%)					
None	19	(22.9)	199	(77.7)	<0.001 *
Yes	64	(77.1)	57	(22.3)	
Education year, N (%)					0.969
≤9	40	(48.2)	124	(48.4)	
>9	43	(51.8)	132	(51.6)	
Alcohol, N (%)					
None	28	(33.7)	130	(50.8)	0.007 *
Yes	55	(66.3)	126	(49.2)	
BQ Chewing, N (%)					
None	24	(28.9)	172	(67.2)	<0.001 *
Yes	59	(71.1)	84	(32.8)	
Cigarette, N (%)					
None	13	(15.7)	69	(27.0)	0.037 *
Yes	70	(84.3)	187	(73.0)	
CYP26A1 SNPs					
rs2068888, N (%)					
A/A	53	(63.9)	172	(67.2)	0.009 *
G/A	23	(27.7)	80	(31.3)	
G/G	7	(8.4)	4	(1.6)	
rs4418728, N (%)					
G/G	7	(8.4)	4	(1.6)	0.009
G/T	23	(27.7)	80	(31.3)	
T/T	53	(63.9)	172	(67.2)	

^a^ Significant difference was test by Chi-square analysis (*p* < 0.05). ^b^ May not total 100% due to rounding. ^c^ Significant difference was test by Independent t-test (*p* < 0.05). SD: standard deviation. BQ: betel quid. * *p* < 0.05.

**Table 2:** Risk factors of oral and pharyngeal cancers for environmental parameters and genetic variant.

Parameters	OR (95% CI) ^a^	OR (95% CI) ^b^
Age group		
age ≤ 50	1	1
age > 50	10.3 (5.6–19.2)	7.6 (2.9–20.0) *
Marriage		
No	1	1
Yes	11.7 (6.5–21.2)	7.5 (3.0–18.9) *
Alcohol		
No	1	1
Yes	2.0 (1.2–3.4)	1.1 (0.4–2.8)
Betel		
No	1	1
Yes	5.0 (2.9–8.7)	8.6 (3.2–23.0) *
Smoking		
No	1	1
Yes	6.84 (1.0–3.8)	2.0 (0.7–5.9)
CYP26A1 rs2068888		
Genotype		
A/A+G/A	1	1
G/G	5.8 (1.7–20.3)	5.0 (1.1–22.1) *

^a^ Logistic regression was used to examine association between risk of oral and pharyngeal cancers and environmental factors and genetic SNP in a single parameter model. ^b^ Logistic regression was used to examine association between risk of oral and pharyngeal cancers and environmental factors and genetic SNP in a *multivariable* model. * *p* < 0.05.

**Table 3:** Attribute risk for genetic Single Nucleotide Polymorphism (SNP) and betel quid use for oral and pharyngeal cancers.

		Controls		Oral and Pharyngeal Cancers						
Risk Allele	BQ Use	n	(%)	n	(%)	OR (95% CI)		AF-Exp (%)	AF-Pop (%)	Synergy Index (95% CI)
0	No	170	(66.4)	21	(25.3)	1		---	---	
0	Yes	82	(32.0)	55	(66.3)	5.4	(3.1–9.6)	81.5	54.0	
1	No	2	(0.8)	3	(3.6)	12.1	(1.9–76.9)	91.7	3.3	
1	Yes	2	(0.8)	4	(4.8)	16.2	(2.8–93.8)	93.8	4.5	0.98 (0.60–1.10)

ORs with 95% CIs and their *p* values were estimated after adjusted for covariates using a multinomial logistic regression model. AF-Exp ([OR − 1]/OR): attributable fraction (percent) among exposed cases. AF-Pop (AF-Exp × % among cases): attributable fraction (percent) among all cases in the population. The synergy index is a test of additive interaction that provides evidence that combined exposures are either super-additive (synergy index > 1), compatible with additive (synergy index = 1), or less than additive (synergy index < 1). The synergy index was calculated as (odds ratio for genetic risk score and betel use − 1) ÷ ([odds ratio for genetic risk score + odds ratio for betel quid (BQ) use] − 2) after adjusted for covariates.

## Supplementary Materials

The following are available online at https://www.mdpi.com/2075-4418/10/11/982/s1. Figure S1: Expression of CYP26A1 between oral and pharyngeal cancer sites. Figure S2: The levels of CYP26A1 mRNA expression in the Different genotypes of rs2068888 (n = 18). Figure S3: The hypothesis for BQ dependent pathogenic effect of CYP26A1 on the occurrence of oral and pharyngeal cancers. Table S1: Distribution of oral and pharyngeal cancer patients (n = 37) associated with characteristics of selected demographic factors, substance use habits, and clinical factors.
