# Supplementary material for: CYP26A1 Is a Novel Biomarker for Betel Quid-Related Oral and Pharyngeal Cancers
Source: Diagnostics (Basel). 2020 Nov 21;10(11):982. doi: 10.3390/diagnostics10110982 (PMC7700252; doi:10.3390/diagnostics10110982)
Supplement: Supplementary file 1 [file diagnostics-10-00982-s001.pdf]

Table S1. Distribution of oral and pharyngeal cancer patients (n = 37) associated with characteristics of selected demographic factors, substance use habits, and clinical factors.

Characteristics	Oral and pharyngeal cancer patients			
	Total	Oral (n = 24)	Pharynx (n = 13)	*p*
Demographic factors				
Male, N (%)		23 (95.8)	12 (92.3)	
Diagnostic age (yrs), mean ± SD	53.5 ± 8.8	52.4 ± 8.3	55.6 ± 9.5	0.166[a]
Ethnicity (n, %)				
Non-Hokkien	6 (19.4)	4 (19.0)	2 (20.0)	1.000[b]
Hokkien	25 (80.6)	17 (81.0)	8 (80.0)	
Educational level (yrs), (n, %)				
>9	9 (29.0)	7 (33.3)	2 (20.0)	0.677[b]
<=9	22 (71.0)	14 (66.7)	8 (80.0)	
Alcohol drinking status, (n, %)				
Non-drinkers	6 (19.4)	3 (14.3)	3 (30.0)	0.358[b]
Drinkers	25 (80.6)	18 (85.7)	7 (70.0)	
BQ chewing status, (n, %)				
Non-chewers	5 (15.2)	2 (8.7)	3 (30.0)	0.149[b]
Chewers	28 (84.8)	21 (91.3)	7 (70.0)	
Age at starting chewing (yrs), mean ± SD	22.6 ± 6.2	23.1 ± 6.4	21.4 ± 5.8	0.540[a]
Years of BQ chewing (yrs), mean ± SD	31.9 ± 10.0	30.1 ± 9.9	36.7 ± 9.4	0.193[a]
Average amount of chewing (quid/day), mean ± SD	26.0 ± 13.6	26.2 ± 14.4	25.7 ± 12.4	0.878[a]
Cumulative lifetime BQ use (pack-years), mean ± SD	81.0 ± 44.1	78.1 ± 47.8	88.3 ± 34.6	0.506[a]
Cigarette smoking status, (n, %)				
Non-smokers	2 (7.4)	2 (10.5)	0 (0.0)	1.000[b]
Smokers	25 (92.6)	17 (89.5)	8 (100.0)	
Clinical factors				
Stage (n, %)				
I+II+III	17 (54.8)	8 (44.4)	9 (69.2)	0.171[c]
IV	14 (45.2)	10 (55.6)	4 (30.8)	
Morphologic type (n, %)				
SCC	32 (86.5)	21 (87.5)	11 (84.6)	1.000[b]
Other carcinoma	5 (13.5)	3 (12.5)	2 (15.4)	
Treatment modality (n, %)				
Surgery	20 (67.6)	15 (62.5)	5 (38.5)	0.161[c]
Surgery + Radiation therapy	17 (32.4)	9 (37.5)	8 (61.5)	

[a]*p* value was calculated from non-parametric Mann-Whitney *U* test. [b]*p* value was calculated from Fisher's exact test. [c]*p* value was calculated from chi-squared (χ^2).

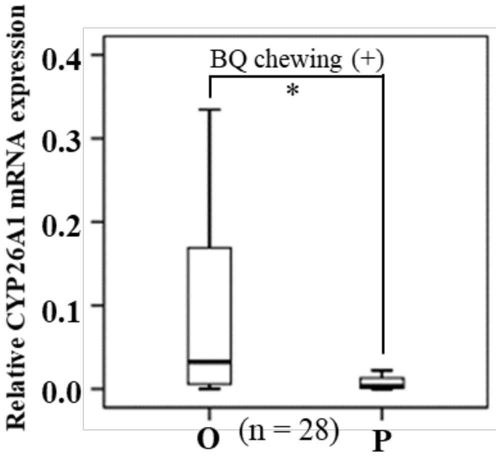

Figure S1. Expression of CYP26A1 between oral and pharyngeal cancer sites. In betel quid (BQ) chewers (n = 28), the relative fold change was estimated using the $2^{-\Delta\Delta Ct}$ method in the oral sites compared with the pharynx sites. The results are represented as the median (IQR). The boxes denote the interquartile ranges, while the bars represent the highest and lowest values, excluding outliers and extreme outliers. *p < 0.05 compared to the normal group.

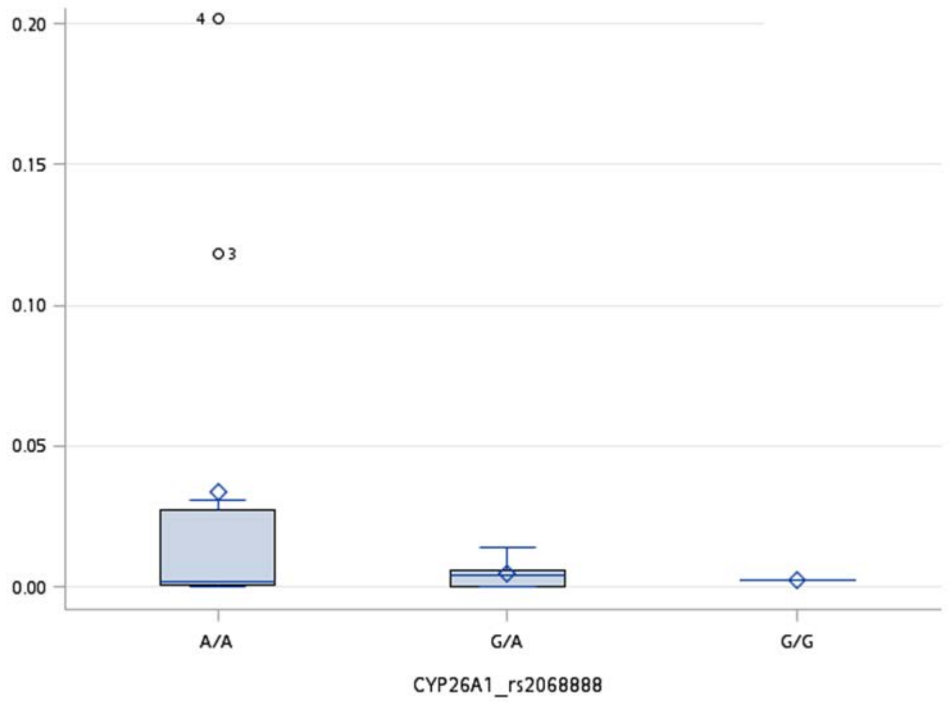

Figure S2. The levels of CYP26A1 mRNA expression in the Different genotypes of rs2068888 (n = 18).

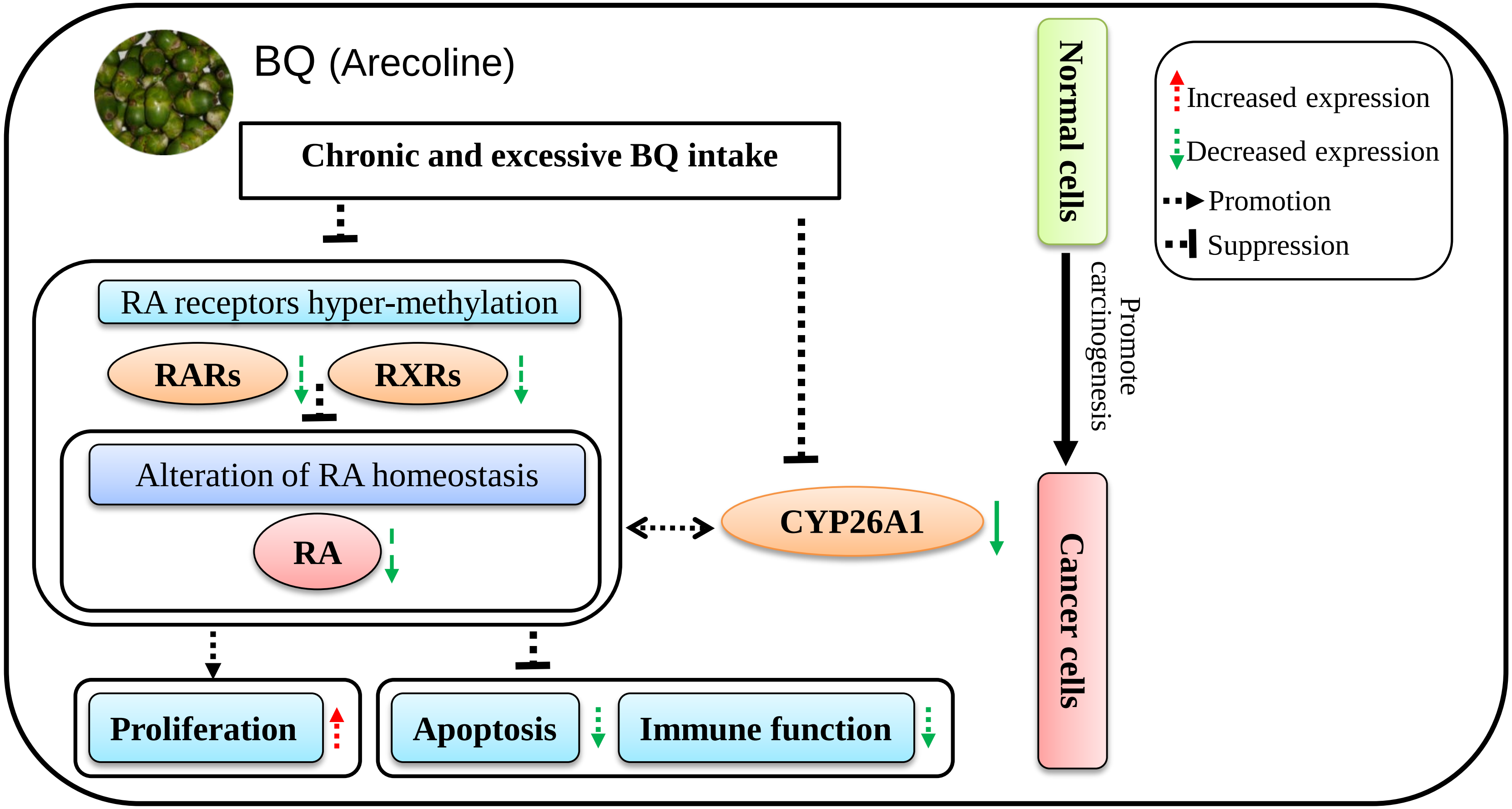

Figure S3. The hypothesis for BQ dependent pathogenic effect of CYP26A1 on the occurrence of oral and pharyngeal cancers
